# What Causes Partial F1 Hybrid Viability? Incomplete Penetrance versus Genetic Variation

**DOI:** 10.1371/journal.pone.0001294

**Published:** 2007-12-12

**Authors:** Hernán López-Fernández, Daniel I. Bolnick

**Affiliations:** 1 Section of Ecology, Evolutionary Biology and Systematics, Department of Wildlife and Fisheries Sciences, Texas A&M University, College Station, Texas, United States of America; 2 Section of Integrative Biology, University of Texas at Austin, Austin, Texas, United States of America; Ecole Normale Supérieure de Lyon, France

## Abstract

**Background:**

Interspecific hybrid crosses often produce offspring with reduced but non-zero survivorship. In this paper we ask why such partial inviability occurs. This partial inviability could arise from incomplete penetrance of lethal Dobzhansky-Muller incompatibilities (DMIs) shared by all members of a hybrid cross. Alternatively, siblings may differ with respect to the presence or number of DMIs, leading to genotype-dependent variation in viability and hence non-Mendelian segregation of parental alleles in surviving F1 hybrids.

**Methodology/Principal Findings:**

We used amplified fragment length polymorphisms (AFLPs) to test for segregation distortion in one hybrid cross between green and longear sunfish (*Lepomis cyanellus* and *L. megalotis*). Hybrids showed partial viability, and twice as much segregation distortion (36.8%) of AFLPs as an intraspecific control cross (18.8%). Incomplete penetrance of DMIs, which should cause genotype-independent mortality, is insufficient to explain the observed segregation distortion.

**Conclusions/Significance:**

We conclude that F1 hybrid sunfish are polymorphic for DMIs, either due to sex-linked DMI loci (causing Haldane's Rule), or polymorphic autosomal DMI loci. Because few AFLP markers were sex-linked (2%), the most parsimonious conclusion is that parents may have been heterozygous for loci causing hybrid inviability.

## Introduction

The evolution of species isolation mechanisms is a central topic in the study of speciation [1]. Postzygotic genetic barriers contribute to the isolation of divergent populations by reducing hybrid fitness and irreversibly preventing introgression regardless of environmental setting. A large effort, both theoretical and experimental, has recently been put into understanding the nature of genetic isolation barriers [2]–[12]. Some of this empirical work provides strong evidence supporting the classic Dobzhansky-Muller model of how genetic barriers might arise [9], [10], [13]. Dobzhansky-Muller incompatibilities (DMIs) result from deleterious epistatic interactions between loci from different parental genomes. When a species of genotype *AABB* is subdivided into two different populations, new mutations independently arise in each population (e.g. *AaBB* or *AABb*), and may eventually fix yielding genetically divergent groups (e.g., *aaBB* and *AAbb*). If the populations subsequently hybridize, interaction between derived alleles *a* and *b* in the hybrids could be deleterious because they evolved in different genetic backgrounds and their compatibility has not been tested by natural selection [3], [7]. Although we illustrate DMIs with two-locus examples throughout the paper, negative epistatic interactions do not have to be limited to pairs of loci. Whether epistasis occurs between pairs or larger sets of genes, DMIs among derived alleles can make the hybrid offspring sterile or inviable, acting as effective genetic barriers between species. The strength of these barriers is expected to increase with divergence time. This is because the expected number of hybrid incompatibilities follows a so-called “snowball effect”, increasing much faster than linearly with the number of derived alleles separating the parental species. Assuming DMIs involve only two loci, the number of expected hybrid incompatibilities increases with the square of the number of non-shared derived alleles, but if more than two loci are involved this rate grows much faster [3], [7].

The classic Dobzhansky-Muller model outlined above tends to assume that the diverging populations have been isolated long enough for the derived genotypes to become fixed (*aaBB* and *AAbb*). In this case, all F1 hybrid offspring will have identical autosomal genotypes *AaBb*, and should have similar viability or sterility. However, empirical observations of hybrid inviability among F1 offspring in animals reveal that many hybrid crosses are only partially viable (i.e. a portion of F1 individuals survive; Table 1. Because the terms viability and inviability can have different meanings in different contexts, throughout this paper we use “partially viable” and “partially inviable” interchangeably to describe crosses in which the percentage of surviving F1 hybrids is significantly less than 100% but greater than 0%). This partial viability of hybrid offspring can be explained in two general ways. First, variation in fitness among F1 siblings might represent incomplete penetrance of DMIs that are present in all individuals. Even though all offspring have identical genotypes with deleterious DMIs (*AaBb*), some hybrid individuals survive because their DMIs are not phenotypically expressed. Variation in hybrid mortality will then be genotype-independent, in the sense that the individuals that survive are genetically indistinguishable from those who die. Alternatively, partial viability might reflect genetic differences among siblings. For example, if one of the interacting loci is sex-linked (e.g., X_a_X_a_BB*X_A_Ybb) and incompatibilities are recessive, then sex-biased mortality may contribute to partial inviability (i.e., *X_A_X_a_Bb* females are viable, but *X_a_YBb* males are inviable), known as Haldane's Rule [1], [8], [14], [15]. Alternatively, if incompatible autosomal alleles have not yet reached fixation in one or both hybridizing parents (e.g., AaBB*AAbb) then a given cross may contain hybrids with and without a given incompatibility (e.g., hybrid genotypes include *AABb* and *AaBb,* with only the latter being inviable because the derived alleles *a* and *b* did not evolve in the same genetic background and may be incompatible). Haldane's Rule and heterozygosity of autosomal DMIs are both specific instances of genotype-dependent partial viability. We emphasize that incomplete penetrance, Haldane's Rule, and heterozygosity of autosomal DMIs may simultaneously contribute to partial viability in a given hybrid cross. In this paper, we wish to determine whether incomplete penetrance alone is sufficient to explain partial viability in hybrid sunfish (Centrarchidae), or whether genotype-dependent mechanisms must also be invoked.

**Table 1:** Variation in F1 viability among different animal crosses.

Taxon	Number of crosses	Complete viability (%)	Partial viability (%)	Complete inviability (%)
Butterflies (one-direction crosses)*^a^*	105	58 (55.0)	24 (22.9)	23 (22.1)
Butterflies (reciprocal crosses)*^a^*	56	35 (62.5)	21 (37.5)	0
Fish (Centrarchidae)*^b^*	35	4 (11.4)	26 (74.3)	5 (14.3)
Frogs (Egg hatching rate)*^b^*	106	3 (2.8)	85 (80.1)	18 (17.0)
Frogs (Larvae metamorphose rate)*^c^*	89	4 (4.5)	49 (55.1)	36 (40.4)
Birds*^d^*	407	357 (87.7)	14 (3.4)	36 (8.8)

See references listed below for details on how viability was estimated in each case. Data for this table were obtained from studies focused on postzygotic isolation and not on hybrid inviability; as a result, we could not include examples in which inviability and hybrid infertility were combined into a unified metric of isolation [50], [51]. Cases listed in this table should be taken as examples of partial inviability and not as an exhaustive review of known cases. Many cases (e.g., butterflies) involve partial inviability arising from Haldane's Rule. Because different studies use different methods to quantify inviability, values across studies are not necessarily comparable. Data from each source are as follows *a*: “Inviability index” data from Presgraves [52]. *b*: mean hybrid viability from supplemental material to Bolnick et al. [41] and see Bolnick & Near [17]. *c*: Based on “Percentage of embryos hatched” and “percentage of larvae metamorphosed” from Sasa et al. [53] (Appendix). *d*: Based on viability categories from supplemental data to Price and Bouvier [54]; estimates of partial viability and complete inviability are based on crosses in categories 3.5 to 5; crosses in categories 1 to 3 vary in degree of hybrid fertility but were considered completely viable for the purpose of this summary.

Both Haldane's Rule and heterozygosity of autosomal DMIs will lead to segregation distortion in the surviving siblings, with an excess of ancestral alleles relative to Mendelian expectations. For example, in a cross of heterozygous parents AaBB*AAbb, surviving offspring with *AABb* genotypes will be more common than their inviable *AaBb* siblings. This will lead to an excess of allele *A* in the surviving offspring. With Haldane's Rule, this distortion will only be seen for sex-linked loci. In contrast, partial viability due to incomplete penetrance leads to genotype-independent mortality and no distortion. Thus, segregation distortion in surviving F1 hybrids can demonstrate genotype-dependent partial viability, suggesting that incomplete penetrance alone is an insufficient explanation. If segregation distortion is found, one can then distinguish between hemizygous (Haldane's rule) and heterozygous DMIs by evaluating whether the distorted loci are sex-linked.

We tested for segregation distortion of amplified fragment length polymorphisms (AFLPs) [16] in one partially viable interspecific cross between the green and longear sunfishes, *Lepomis cyanellus* and *L. megalotis* [17]. AFLPs have previously been used to detect segregation distortion, as they allow one to scan large numbers of loci even in non-model organisms [18], [19], [20], [21]. Hybrid mortality can lead to a deficit or excess of a given AFLP marker depending on whether the marker is linked to the lethal allele or to the homologous non-lethal allele, and depending on which parent carries the marker and the DMI allele. Additionally, we use AFLPs to determine how many AFLP markers are linked to sex in the green sunfish.

## Results

### Measures of cross viability

**Figure 1:**
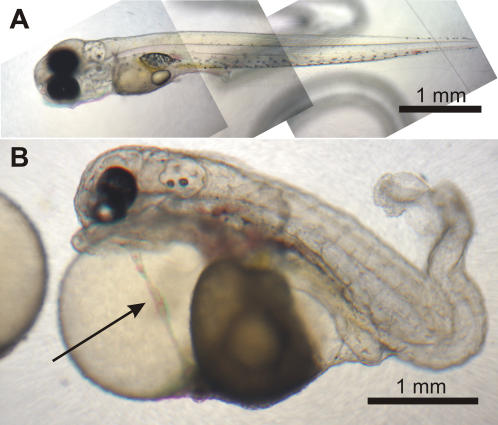
F1 hybrids between a female *Lepomis cyanellus* and a male *L. megalotis* approximately six days after hatching, just before the swimming stage. Scale bars are approximate. A. Morphologically normal larva; notice the elongate and straight spine and the comparatively reduced size of the body cavity. B. Morphologically deformed sibling of the fish in A; the spine is variously kinked and twisted, especially on the distal third, and the body cavity is notoriously enlarged. The arrow indicates the heart morphology, which appears analogous to that of the *heartstrings* developmental mutation observed in zebrafish (see discussion and [43], Fig. 1E). Approximately two thirds of the hybrid offspring described in this study showed morphological abnormalities of the types observed in this example (Table 2).

Counts of egg and larval survivorship at different stages of development revealed large differences between the intraspecific cross and the hybrid sunfish crosses (Table 2). Although fertilization rates were very similar between crosses (well over 90%, Table 2), hybrid hatching rates were 40% of the green sunfish hatch rates. Furthermore, while all green offspring were morphologically normal and survived to the free-swimming stage, almost two thirds of the hybrid hatchlings exhibited visible morphological abnormalities (Fig. 1). Nearly a quarter of the hybrid hatchlings died by the time they should have started swimming (about 7 days after hatching). Although no deformed fry survived long after free-swimming, the morphologically normal hybrid fry were able to develop to sexual maturity. For comparison, the one other published cross between green and longear sunfish found the viability of hybrids was 110% of the control cross viability [22]. However, this cross used a northern population of longear, which has since been reclassified as a distinct species from the southern *L. megalotis* used here [23]. In other *Lepomis* crosses, different studies have yielded moderately repeatable measures of hybrid viability (e.g., *L. gulosus*×*L. macrochirus* mean viability = 35%, stdev = 22, N = 5 crosses; *L. macrochirus*×*L. gulosus* mean viability = 77%, stdev = 19.5, N = 4 [17]).

**Table 2:** Viability of green and hybrid crosses at different points of egg and larval development.

Cross	Total eggs	Fertilized eggs (%)	Total hatched eggs (%)	Hatched normal (%)	Alive at swimup (%)
Green: *L. cyanellus*	754	729 (96.7)	656 (90)	656 (100)	656 (100)
Hybrid: *L. cyanellus*×*L. megalotis*	2189	2075 (94.7)	756 (36.4)	283 (37.4)	221 (78.1)

### AFLPs and non-Mendelian segregation in the green and hybrid crosses

AFLP analysis of seven primer pairs for the intraspecific cross of *Lepomis cyanellus* (green cross) resulted in 768 scored loci, of which 429 were polymorphic. Eighty one (18.8%) of the polymorphic loci deviated significantly from Mendelian expectations. By applying an α = 0.05 for each allele, we would expect roughly 5% of tests to exhibit non-Mendelian segregation by chance alone. The additional segregation distortion likely reflects experimental and scoring error as this is an intraspecific cross [18], [24]. Unexpected allele frequencies can also arise from homoplasy, in which multiple independent loci yield identical fragment lengths. Such homoplasy is not expected to bias comparisons between green and hybrid crosses. Intraspecific segregation distortion provides a benchmark for comparison with distortion in hybrids.

AFLP analysis of the hybrid cross between *L. cyanellus* and *L. megalotis* resulted in 764 scored loci of which 581 were polymorphic and 214 (36.8%) deviated from Mendelian expectations. A Chi-square test confirmed that the segregation distortion in hybrids was significantly higher than in their half-siblings in the green cross (*X* ^2^ = 38.46, d. f. = 1, p<0.0001). As both crosses shared the same female parent and we used the same primer pairs and scoring method, the excess 17.9% segregation distortion in the hybrid cross demonstrates that incomplete penetrance is insufficient to explain partial hybrid viability in green×longear sunfish hybrids. We conclude that there is genetic variation underlying the variation in survival among full-sibling hybrids. A few other studies have identified genetic variation in F1 hybrid viability [8], [19], [20], [25], though this has not previously been used to address the question of partial hybrid viability.

### AFLP results and sex-linkage in the green cross

We reared 27 *Lepomis cyanellus* to sexual maturity, yielding 14 females and 13 males (three of the 30 genotyped individuals were lost to disease before maturity). Five (1.2%) of the 429 polymorphic AFLP loci scored for the green cross were found to be significantly linked to sex. Two additional loci were strongly biased towards one sex but not significant after Bonferroni correction (Table 3). Only one fragment (0.23% of sampled loci) was diagnostic for one sex, being present in all females and none of the males (p<0.0005, Table 3, 202 bp long). These low rates of sex-linkage are not surprising given that there is no karyotypic evidence for dimorphic sex chromosomes in *Lepomis.*

**Table 3:** AFLP loci linked to sex in 14 female and 13 male individuals of *Lepomis cyanellus* (25 offspring and parents from green cross).

Primer pair	Locus size (base pairs)	Females with allele (percent, n = 14)	Males with allele (percent, n = 13)	χ^2^	p-value
3	202	14 (100)	0 (0)	27	2.7*10^−7^***
	223	1 (7)	12 (92)	19.58	9.6*10^−6^**
4	97	13 (93)	1 (8)	19.58	9.6*10^−6^**
	105	12 (86)	2 (15)	13.35	2.6*10^−4^ NS
	134	12 (86)	1 (8)	16.4	5.0*10^−5^*
	135	2 (14)	12 (92)	16.4	5.0*10^−5^*
	238	9 (64)	0 (0)	12.54	4.0*10^−4^ NS

* = p<0.05, ** = p< 0.005, *** = p<0.0005

Significance values were obtained from chi-square tests with sequential Bonferroni correction for 429 polymorphic AFLP alleles.

## Discussion

Hybrid offspring between *Lepomis cyanellus* and *L. megalotis* are only partially viable. Despite very similar rates of fertilization in the green and hybrid crosses, hatching rates were much lower in hybrids and produced a majority of deformed larvae that were largely unable to survive to the free-swimming stage (Table 2). Nonetheless, a portion of hybrids showed no apparent morphological abnormalities, survived well after free-swimming (Table 2), and were able to develop to maturity (D. I. Bolnick & H. López-Fernández, unpubl. observations). Partial viability of hybrid offspring such as observed in *Lepomis* is a widespread outcome of crosses among a variety of animal groups (Table 1).

We found significantly larger segregation distortion of AFLP inheritance in the hybrid cross in comparison to the intraspecific cross. Because incomplete penetrance of DMI loci produces genotype-independent deleterious effects, it cannot account for the increased genotypic bias detected in the hybrids. Although incomplete penetrance may still contribute to the partial viability observed here, genotype-dependent mechanisms are required to account for excess segregation distortion. As mentioned above, one cross is sufficient to discard incomplete penetrance as the sole cause of partial inviability, but it remains to be determined whether repeated crosses would exhibit the same amount of segregation distortion, and whether the same loci exhibit segregation distortion in different crosses.

### Haldane's rule

Haldane's rule can cause segregation distortion only in proportion to the number of markers that are linked to sex. Taxa with small hemizygous sex-linked regions of the genome are expected to exhibit little if any sex-biased hybrid sterility or inviability [26]. In green sunfish, analysis of sex-linked AFLP alleles revealed a remarkably low number of potentially sex-linked loci. Less than two percent of all AFLP markers in the green cross were significantly associated with sex. Only locus 202 is restricted to all individuals of one sex (female) and is absent from all individuals of the other, thus being the best candidate for a real sex-linked marker (Table 3). It should be noted that this locus showed no segregation distortion. Loci that are either present only in some individuals of one sex (e.g. locus 238, Table 3) or biased towards one sex but not absent from the other (e.g. loci 97, 134) may either be false-positives, or may be loci that are on the same chromosome as a sex-determining region, but undergo low rates of recombination with sex loci. Given the relatively uniform size of the centrarchid chromosomes [26], we would expect roughly 4% (18) of the AFLP fragments to be completely linked to sex if there was an entirely non-recombining sex chromosome. Because only one marker (0.23% of the markers) was diagnostic for sex, we posit that sex determination in *Lepomis* results from sex-loci rather than hemizygous chromosomes.

Our genetic results are congruent with cytological studies of *Lepomis* and a number of other centrarchid genera, which have been unable to distinguish karyotypically distinct sex chromosomes [27]. Because karyotypic analyses have been uninformative, little is known about sex determination in centrarchids. Gomelsky et al. [28] found evidence for heterozygous-male sex determination in the Black Crappie (*Pomoxis nigromaculatus*), based on sex ratios of offspring from hormonally sex-reversed males crossed with normal females. In contrast, the only AFLP marker that was perfectly linked with sex in our study was found in females, suggesting that females may be the heterozygous sex in *Lepomis cyanellus.* Fishes are well known for divergent sex determination systems between even closely related species [29]. It will therefore be useful to conduct parallel sex-linkage studies in other *Lepomis* species in the future.

The apparent lack of chromosomal sex determination in centrarchids implies a reduced role for Haldane's rule in genetic isolation of lineages within the clade. A lack of sex chromosomes would limit the DMIs in centrarchids to autosome-autosome interactions, which are of smaller effect than sex-autosome interactions [7], [26], [30], [31], and which tend to be recessive [11]. Theory predicts that large-effect DMIs should emerge from interactions between dominant autosomal alleles and recessive sex-linked alleles, and that the number of DMIs should increase with the size of the sex-linked region because more hemizygous recessive alleles can interact deleteriously with autosomal loci [8], [26], [30]. Because there is little or no hemizygous sex-linked region in *Lepomis*, there is little opportunity for deleterious interactions with autosomal loci. In conclusion, our findings support Bolnick & Near's [17] hypothesis that Haldane's rule is absent or weak in centrarchids and does not play a major role in the evolution of genetic barriers within the clade. Clearly, less than 2% association with sex is not enough to produce the 18% excess segregation distortion found in the hybrid cross through Haldane's rule.

### Parental heterozygosity of Dobzhansky-Muller incompatibilities

The partially viable hybrid cross showed twice as much AFLP segregation distortion than the intraspecific green cross (Table 3). Incomplete penetrance of lethal epistasis is not expected to bias genotype frequencies in this way, and too few alleles are sex-linked to invoke Haldane's Rule. An alternative cause of genotype-dependent mortality invokes polymorphism for autosomal DMIs among the sibling F1 hybrids. Such polymorphism requires that one or both parents be heterozygous for DMI loci. In the two-locus Dobzhansky-Muller model of hybrid incompatibility, the heterozygote genotype *AaBB* is a necessary step on the way to fixation of the genotype *aaBB* in one of the diverging populations. At any given time before fixation, there will be individuals in each population that possess each possible genotype (e.g. *AABB*, *AaBB*, *aaBB*). Hybrids of heterozygous parents from these populations will have a mixture of ancestral and derived alleles that may experience Dobzhansky-Muller interactions with varying phenotypic effects. As populations continue to diverge, an increasing number of loci may be involved in DMIs, all of which must pass through a period of polymorphism. Such polymorphic populations for DMIs have been previously reported for a number of organisms such as plants in the genus *Crepis* [32], *Drosophila* [33], [34], *Tribolium* beetles [35], [36], *Chorthippus* grasshoppers [37], and *Nasonia* parasitoid wasps [38]. By process of elimination, we speculate that similar polymorphism for DMIs explains the genotype-dependent partial viability documented in this paper. We predict that future replicate green×longear sunfish crosses will also identify partial viability, but with varying degree of segregation distortion and involving different combinations of distorted loci.

Our results suggest that a surprisingly large number of loci are linked to genes involved in hybrid inviability. Many individual hybrids will thus be likely to carry multiple DMI loci, and most individuals should carry at least a few such loci. The moderate hybrid survivorship suggests that many DMIs are individually insufficient to cause mortality. The present study does not allow us to determine whether mortality in these hybrids is a consequence of interactions among many loci, or increasing probability of mortality with increasing numbers of independent DMIs. It is intriguing to note that hybrid mortality was not restricted to a single developmental stage, ranging from early embryos to hatching, and subsequent swim-up. This diversity of mortality stages suggests that there may be a wide diversity of DMIs operating in different individuals. Variation in the degree and timing of DMI effects may be widespread.

Hybrid viability, sex ratio, and deformity rates varied among different crosses of laboratory strains of *Tribolium freemani* with *T. castaneum* [36]. Reed & Markow [34] demonstrated that intraspecific genetic polymorphism in *Drosophila mojavensis* caused variable degrees of hybrid male sterility in crosses with *D. arizonae*. These studies and our own results highlight the complex genetic basis of hybrid inviability, with phenotypic effects ultimately depending on the specific alleles involved in negative epistatic interactions in each hybrid individual. Consequently, the degree of post-mating isolation between diverging populations may frequently depend on the individuals selected for a given cross.

Genetic polymorphism within diverging lineages is usually thought to characterize the early stages of speciation, when genetic postzygotic incompatibility is not yet completely established [34], [37]. When compared to *Lepomis* and other centrarchid fishes, previously reported heterozygosity for DMI loci has generally been found between populations that have diverged relatively recently. For example, *Chorthippus* grasshoppers have diverged for approximately 0.5 MY [37] and *Nasonia* wasps for 0.1 to 0.2 MY [39]. In contrast, DMI loci causing segregation distortion in the hybrids between *Lepomis cyanellus* and *L. megalotis* are not completely fixed in these species despite an estimated 13–16 MY since both species diverged from their common ancestor [40]. Furthermore, hybrid crosses between even older centrarchid lineages also produce partially viable hybrids [17], suggesting that DMI polymorphisms may be widespread within the clade.

An interesting point for speculation is whether the observed polymorphisms are due to recent mutations that have not had time to go to fixation, or if they result from very slow fixation of much older derived alleles. If DMI alleles tend to fix quickly (e.g., due to selection), we would be unlikely to see polymorphism at any randomly chosen point along 13–16 MY history of divergence. The possible polymorphism documented here suggests that fixation of DMIs may instead be quite slow, implying roughly neutral fixation of alleles. This is consistent with comparative evidence suggesting very slow accrual of hybrid inviability in centrarchids [17], though there is some evidence that divergent selection may accelerate fixation of DMIs [41], [42].

There are both caveats and unforeseen aspects of our results that suggest further avenues for research. First and foremost, our conclusions are based on a single experimental hybrid cross. Although our present results are clear-cut, repeating the analyses with a larger number of crosses would be valuable. More interestingly, replicate crosses would serve to address the slightly different question of how the degree of hybrid incompatibilities varies among crosses. Often, variation in hybrid viability between replicate crosses is treated as experimental noise, whereas our results suggest that parental populations contain segregating variation for DMIs. Hence it is very likely that replicate crosses, even using the same source populations, will reveal different loci involved in segregation distortion or different levels of viability. Much could be learned about the nature and phenotypic effects of DMIs in centrarchids by comparing the patterns of segregation distortion among replicate crosses. Another clear avenue for research involves the developmental timing and effects of DMI loci on hybrid offspring. Patterns of segregation distortion may depend on the stage at which we sample hybrid offspring, if certain types of inviability tend to act at specific developmental stages.

Some of the morphological deformities observed in the hybrids may be particularly amenable for both locating the genetic loci of DMIs in centrarchids and studying the specific phenotypes of incompatibility. For example, many deformed hybrid sunfish (Fig. 1B) showed a phenotype that is very similar to the mutated *heartstrings* morphology described for zebrafish. This syndrome allows normal development of the heart during early development, but later the heart fails to loop and remains elongate and “string-like” (thus the name). Eventually circulation ceases and the individual dies. In zebrafish this condition is caused by a homozygous recessive mutation in the T-box family transcription factor *tbx5.* A similar mutation in *tbx5* also causes heart syndromes in mice and humans [43]. If a relationship between the *heartstrings* syndrome and centrarchid DMIs is found, it would provide the first evidence of a DMI locus in vertebrates.

### Summary

In conclusion, siblings from F1 hybrid crosses among sunfish (*Lepomis*) exhibit variable fitness. In our single experimental cross, we found that this partial viability is genotype-dependent, which cannot be attributed to incomplete penetrance. Given the low frequency of sex-linked loci, we hypothesize that this genetic variation reflects parental polymorphism at loci involved in intrinsic genetic incompatibilities. The large number of such loci is consistent with the variable stage at which mortality occurs in hybrid siblings. Theory suggests that long-diverged taxa should exhibit large numbers of DMIs [44], though it is surprising that so many of them appear to be polymorphic in *Lepomis*. We suggest that studies of taxa exhibiting partial hybrid inviability will prove particularly fruitful in identifying genes involved in post-mating isolation. This has certainly been true for studies of Haldane's Rule, which is one of the most illuminating and widespread patterns of reproductive isolation [1].

## Materials and Methods

### Adult specimens and crosses

We performed one interspecific cross between a female green sunfish (*Lepomis cyanellus*) and a male longear sunfish (*L. megalotis*), and one intraspecific control cross using the same green sunfish female and a green sunfish male. A single cross is sufficient to evaluate whether incomplete penetrance alone explains partial inviability, though additional crosses would be needed to extend our conclusions to the entire population or species. Adult fish were caught from wild populations in Waller and Shoal creeks in Austin, Texas during the spring 2005 breeding season. These species span the oldest divergence within the genus *Lepomis* (13–16 million years: [40]) and share the same number of chromosomes (2n = 48: [27]). A localized population of green sunfish with 2n = 46 was reported for West Virginia [27], but extensive karyotyping of Texas populations revealed a fixed number of chromosomes of 2n = 48 (J. Gold., Pers. Comm.). AFLP analysis of multiple individuals in each species revealed no evidence of introgression in the populations used for the crosses.

Following methods described in Childers [45], we performed an intraspecific cross of *L. cyanellus* (“green cross” from here on) and a hybrid cross between *L. cyanellus* and *L. megalotis* (“hybrid cross” from here on). The crosses were performed simultaneously, dividing a batch of eggs from one green sunfish female into two Petri dishes and fertilizing each with sperm from different males. This resulted in two sets of fry from the same green sunfish dam but sires from different species. Fin clips of both parents were taken and preserved in ethanol 95% at −80°C for genetic analysis. Limited availability of wild-caught reproductively mature female *Lepomis megalotis* prevented us from performing intraspecific crosses within *L. megalotis*, and interspecific crosses between female *L. megalotis* and male *L. cyanellus.*

Fertilized eggs from both crosses were incubated at 23°C in Petri dishes filled with water treated with 2 drops per gallon of 2% methylene blue to avoid fungal growth and gently aerated with an airstone. Within 24 hours after hatching we preserved 30 larvae from the hybrid cross in ethanol 95% for genetic analysis to detect segregation distortion due to embryonic mortality. Fin clips for genetic analysis were taken from 30 green-cross individuals when they reached a total length of approximately 3 cm. These same individuals were used to test for sex-linked AFLP alleles. The fish were reared to sexual maturity in individual aquaria, at which point we identified their sex by dissection to directly examine gonads. Sampling green sunfish at a later stage of development than the hybrids should not significantly bias our results, because viability in the green cross was so high. Any segregation distortion that does occur in the green cross at the larval stage should also persist into the juvenile stage.

### Measures of offspring viability

To determine offspring viability in both crosses, we counted the initial number of eggs in each cross, and calculated the proportion of eggs fertilized, eggs hatched, deformed hatchlings, and of hatchlings that developed to free-swimming stage. Total number of eggs and number of fertilized eggs were counted approximately 1 to 2 hours after mixing sperm and eggs. Eggs were considered fertilized when two or four cell stage embryos could be identified. Hatching occurred within 30 to 48 hours and counting was done several hours after to ensure that all viable eggs had hatched. During hatching counts we determined the fraction of larvae with visible morphological deformities, from slight kinking of the spine to major deformation of the body cavity and caudal region (H. López-Fernández & D. I. Bolnick, unpubl. observations). We determined the fraction of larvae surviving to swim-up at the time-point when the number of swimming fry asymptoted. Percent values were calculated based on the number of individuals surviving each stage of development. For example, 37.4% of the total hatched hybrid eggs were normal and 78.1% of these survived to swimup (Table 2).The larvae were then transferred to 10-L aquaria for rearing, and later moved into 160-L aquaria.

### AFLP fingerprinting

DNA isolation was performed using the DNeasy extraction kit (Qiagen) with a final elution in 100 µL ddH_2_0. Sequences of adaptor oligos and all other primers used in the AFLP amplification are given in Table 4. Restriction-ligation reactions were performed using a mixture of two adaptor oligos designed specifically for restriction enzymes *MseI* and *EcoRI*. Adaptors for each enzyme were combined in equal volumes, denatured at 95°C for 5 min. in a thermocycler and cooled to room temperature for 10 min. before use. Concentration of oligos was 0.62 g/L for *Mse*I adaptors and 0.064 g/L for *EcoR*I adaptors. For each reaction, 1 µL of each pair of adaptors was combined with 1.1 µL 10× T4 DNA Ligase Buffer (Promega), 0.33 µL T4 DNA Ligase (Promega), 1.1 µL 0.5 M NaCl, 0.55 µL BSA (1 mg/mL), 0.1 µL *MseI* (New England Biolabs), 0.42 µL *EcoRI* (New England Biolabs), 3.9 µL of ddH_2_0, and 1.5 µL of total genomic DNA. Restriction-ligation was performed by incubation at 37°C for 2 hours in a thermocycler, and diluted with 60 µL 1× TE buffer. The entire diluted volume was then cleaned using the QiaQuick PCR cleanup kit (Qiagen) with final elution of the clean product in 120 µL 1× TE buffer.

**Table 4:** Sequences of adaptor oligos, preselective and selective primers used in this study.

	Oligonucleotide sequence
**Adapters**	
*EcoR*I Adaptor 1	CTC GTA GAC TGC GTA CC
*EcoR*I Adaptor 2	AAT TGG TAC GCA GTC TAC
*Mse*I Adaptor 1	GAC GAT GAG TCC TGA G
*Mse*I Adaptor 2	TAC TCA GGA CTC AT
**Preselective primers**	
*EcoRI* Preselective	GAC TGC GTA CCC AAT TCA
*MseI* Preselective	GAT GAG TCC TGA GTA AC
**Selective primers**	
*EcoRI* 1-FAM	GAC TGC GTA CCC AAT TCA CA
*MseI* 1	GAT GAG TCC TGA GTA ACT TA
*MseI* 2	GAT GAG TCC TGA GTA ACT G
*MseI* 3	GAT GAG TCC TGA GTA ACT T
*MseI* 4	GAT GAG TCC TGA GTA ACT C
*MseI* 5	GAT GAG TCC TGA GTA ACT G
*MseI* 6	GAT GAG TCC TGA GTA ACT A
*MseI* 7	GAT GAG TCC TGA GTA ACT CA

Underlined bases indicate the number of selective nucleotides in each primer. See text for protocol details.

Pre-selective primers added one extra base to the adaptor sequences, thus reducing the number of amplified fragments to roughly one quarter of the total restriction fragments obtained in the restriction-ligation reaction. 4 µL of cleaned restriction-ligation product were combined with 10.4 µL ddH2O, 4 µL buffer E (0.3 M Tris base, 75 mM (NH_4_)_2_SO_4_, 7.5 mM Mg_2_Cl_2_ at pH 9.0), 0.5 µL 10 mM dNTPs, 0.5 µL 20 µM *MseI* pre-selective primer, 0.5 µL 20 µM *EcoRI* pre-selective primer, and 0.1 µL of Taq polymerase (Promega) for a final 20 µL reaction. Pre-selective thermocycler conditions were 72°C for 2 min.; 25 cycles of 94°C for 20 s, 56°C for 30 s, and 72°C for 4 min.; and 1 cycle at 60°C for 30 minutes. Pre-selective PCR product was diluted with 40 µL 1× TE buffer.

Selective amplification primers added two or three additional bases to the pre-selective sequences to further reduce the number of amplified fragments (Table 1). We used seven *MseI* selective primers in combination with an *EcoRI* selective primer labeled with FAM fluorescent dye for later fragment detection. For selective amplification we further diluted the pre-selective PCR product 1∶9 in ddH_2_O. Reactions combined 1.5 µL of diluted pre-selective product with 4.2 µL ddH_2_O, 2 µL buffer E (see preselective PCR), 0.25 µL 10 mM dNTPs, 1 µL 5 µM *MseI* selective primer, 2 µL FAM-labeled 1 µM *EcoRI* selective primer, and 0.05 µL Taq (Promega) for a total reaction volume of 11 µL. Thermocycler conditions were 94°C for 20 s; 50 cycles 94°C for 20 s, 66–56°C for 30 s (first cycle at 66°C, decreased by 1 degree per cycle to 56°C degrees, remaining cycles performed at 56°C), 72°C for 2 min; 60°C for 30 min. 1 µL of selective product was mixed with 0.5 µL of ROX size standard (Applied Biosystems) and 8.5 µL Hi-Di Formamide (Applied Biosystems) for fragment detection in an ABI 3100 (Applied Biosystems) genetic analyzer. Restriction-ligation and preselective PCR reactions were performed simultaneously for all samples from a given cross, and selective reactions with each primer pair were performed simultaneously for all individuals.

### AFLP sampling criteria and scoring methods

In the absence of underlying genetic biases, AFLPs should theoretically produce Mendelian segregation of alleles at proportions of 0.5, 0.75 or 1.0 [24], [25]. Deviation from these proportions may be caused during scoring due to variable peak heights across samples, or stutter arising from PCR error (e.g.: [18]). Because we were interested in detecting Mendelian distortion associated with hybrid incompatibility, we aimed at minimizing distortion due to scoring and experimental error. Only peaks with intensities of at least 50 fluorescence units were included in analyses and only bands ranging in size between 50 and 550 base pairs were analyzed. Scoring was performed both automatically and manually, in order to maximize repeatability based on 30 replicate analyses of a single individual for primer pair 1 (see section on Mendelian distortion calculations for details). Manual methods included scoring all observed peaks and a “25% method” in which a peak in the electropherogram was scored as present if its intensity was at least 25% of the intensity of the peaks 1 base pair before and 1 base pair after. Although the 25% cutoff is arbitrary, we found that it yields the highest repeatability of scoring replicate genotypes of a single individual, minimizing scoring error arising from stutter created by addition or subtraction of bases during PCR extension [46]–[48]. Our results are qualitatively the same, whichever scoring method we used, so here we only report the scoring results from the 25% method. We did not observe any bands in the offspring that could not be associated with either parent. All scoring was performed in GeneMarker version 1.3 (Softgenetics) using the software's routine for size-call correction before scoring. Binning was performed using a pre-defined set of fragment sizes based on the profile of both parental individuals in each cross. This pre-defined panel of markers was created for each cross and for each primer pair using the Panel Design program in GeneMarker.

### Expected and observed segregation of alleles in intra- and interspecific *Lepomis* crosses

We were interested in determining whether non-Mendelian segregation in the hybrid cross was higher than in the green cross. We compared AFLP allele frequencies in both crosses against Mendelian expectations for dominant alleles, given parent genotypes. Distortion in the green (intraspecific cross) was assumed to reflect experimental or scoring error. A higher rate of distortion in the hybrid cross indicates genotype-dependent mortality.

Given the dominant nature of AFLPs, when a given AFLP marker was present in both parents, the expected frequencies of the marker in the offspring were either 0.75 or 1.0. Alternatively, when an AFLP locus was present in only one of the parents, the expected frequencies in the offspring were 0.5 or 1.0. Accounting for parental genotype at each AFLP locus, we estimated goodness of fit to Mendelian expectations by calculating the confidence interval of the proportion of individual offspring in which a given allele was present. This calculation uses a finite correction for sampling a binomial population based on the relationship between the *F* distribution and the binomial distribution [49] (Equations 24.28 and 24.29, see below). If *n* is the number of individuals in the population being sampled and *X* is the number of individuals with a particular marker, the estimated frequency of individuals with the marker is 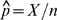. The upper and lower limits of the confidence interval for 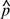 (*L*_1_ and *L*_2_) are
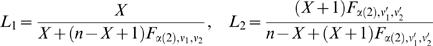
where
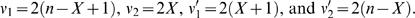
A given AFLP fragment was judged to exhibit segregation distortion if the 95% confidence interval around its estimated frequency 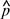 did not include Mendelian expectations of *p* = 0.5 or 1.0 (if the AFLP band was present in only one parent), or *p* = 0.75 or 1.0 (if the AFLP band was present in both parents). We counted the number of polymorphic fragments exhibiting segregation distortion in the green cross, and in the hybrid cross.

In a number of instances, observed allele frequencies were close but not equal to *p* = 1.0, yet were identified as significant segregation distortion. This is because the confidence intervals described above become very small as *p* approaches 1.0. For instance, when a fragment is present in 29 out of 30 individuals, this frequency is clearly inconsistent with an expectation of 100% of individuals having the allele. However, this small deviation could result from scoring error rather than a genuine deviation from Mendelian segregation. To evaluate the rate of scoring error, we determined the rate of false negatives by genotyping a single individual 30 replicate times, using primer pair 1 (Table 2) and the scoring methods described above. We found that of 68 alleles in that individual, 56 (82.4%) were scored as present in all 30 repeats, 9 (13.2%) were scored as present in 29 repeats, and 3 (4.4%) were scored in only 28 repeats. We therefore used an adjusted Mendelian expectation, treating fragment frequencies of *p* = 0.933 (X = 28/30) as being consistent with *p* = 1.0. Consequently, any AFLP fragment whose frequency 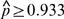 was treated as consistent with Mendelian expectations. This approach is conservative and, if anything, may under-estimate rates of segregation distortion.

We emphasize that some patterns of segregation distortion could not be detected by our method. For instance, consider a case where an AFLP fragment is present in one parent, which may be homozygous or heterozygous, in which case we expect an allele frequency of 1.0 or 0.5 in the offspring. If the parent were actually heterozygous, strong mortality associated with the null allele could lead to an observed frequency of *p* = 1.0 in the surviving hybrids, even though the proper expectation was 0.5. Because we are unable to distinguish between heterozygous and homozygous parents, we would interpret this strong segregation distortion as being consistent with Mendelian expectations. Consequently, our test tends to be conservative and may underestimate true rates of segregation distortion. More robust tests would require co-dominant markers such as microsatellites, which are not available in sufficient number in *Lepomis*.

### Proportion of sex-linked alleles in *Lepomis cyanellus*

To evaluate whether any alleles exhibit sex-biased transmission in the intraspecific green sunfish cross, we sexed mature green sunfish siblings by inspecting gonads via dissection. We then carried out a chi-square test for each locus to look for sex differences in allele frequencies. Because we evaluated 429 polymorphic alleles, we used a Bonferroni correction when evaluating statistical significance (α = 0.0001).
